# Paracoccidioidomycosis manifested by sarcoid-like cutaneous lesions with severe systemic disease: a rare and challenging diagnosis

**DOI:** 10.1590/0037-8682-0605-2023

**Published:** 2024-03-25

**Authors:** Bruna dos Anjos Bortolini, Rachel Bertolani do Espírito Santo, Lucia Martins Diniz, Robson Dettmann Jarske, Natália Tebas de Castro

**Affiliations:** 1 Hospital Universitário Cassiano Antônio de Moraes, Departamento de Clínica Médica, Disciplina de Dermatologia, Vitória, ES, Brasil.; 2 Universidade Federal do Espírito Santo, Vitória, ES, Brasil.; 3 Hospital Universitário Cassiano Antônio de Moraes, Unidade de laboratório de Anatomia Patológica, Vitória, ES, Brasil.

**Keywords:** Paracoccidioidomycosis, Dermatology, Granuloma

## Abstract

Cutaneous involvement in paracoccidioidomycosis (PCM) can exhibit a highly polymorphic spectrum. The infiltrative pattern corresponds to up to 26.6% of observed skin lesions, including sarcoid-like plaques, a rare presentation of cutaneous lesions in PCM. This clinical expression is almost exclusively cutaneous, and its histology reveals a tuberculoid granuloma with a scarcity of fungi, leading to misdiagnosis as other granulomatous diseases. Here, we report a rare form of chronic multifocal paracoccidioidomycosis manifesting as sarcoid-like skin lesions misdiagnosed as granulomatous rosacea in a patient with severe systemic disease.

## INTRODUCTION

Paracoccidioidomycosis (PCM) is a systemic mycosis caused by thermally dimorphic fungi of the genus *Paracoccidioides* ^1^. Typically, most patients have a history of rural living and are frequently associated with smoking and alcohol abuse. PCM is transmitted via inhalation of soil-borne propagules. The outcome, whether spontaneous cure, active disease, or latent disease, depends on the interaction between host immunity and fungal virulence^2^ ^,^ ^3^.

The clinical presentation of paracoccidioidomycosis varies. The primary organs affected are the lungs (90%), mucosa (59.6%), lymph nodes, adrenal glands (50%), nervous system (27%), and skin^4^ ^,^ ^5^ ^,^ ^6^. Cutaneous lesions have been reported in 30-54% of studied patients^7^. These show variable morphology, and may occur as ulcers, papules, nodules, and vegetative, verrucous, or infiltrative plaques. A rare pattern described as infiltrated or lichenoid lesions, namely sarcoid or sarcoidosis-like is also reported^3^ ^,^ ^7^. Usually, this clinical expression is almost exclusively cutaneous, and histology reveals a tuberculoid granuloma with a scarcity of fungi, leading to its misdiagnosis as other granulomatous diseases^1^.

Here, we present a rare cutaneous manifestation of PCM characterized by sarcoid-like skin lesions in a patient with a severe systemic chronic form of the disease. Initially, the condition was misdiagnosed and treated as granulomatous rosacea. This case is noteworthy because of its challenging diagnosis and the rare association of sarcoidosis-like skin patterns with extracutaneous lesions, in addition to being the only report of exuberant systemic manifestations found in the literature (Brief Review: Cochrane Library, LILACS, SciELO, MEDLINE, PubMed, and PubMed Central). This study was submitted in accordance with the ethical standards of the responsible committee** **on** **human experimentation, nº 77/2023/GEP/HUCAM-UFES-EBSERH.

## CASE REPORT

**FIGURE 1:**
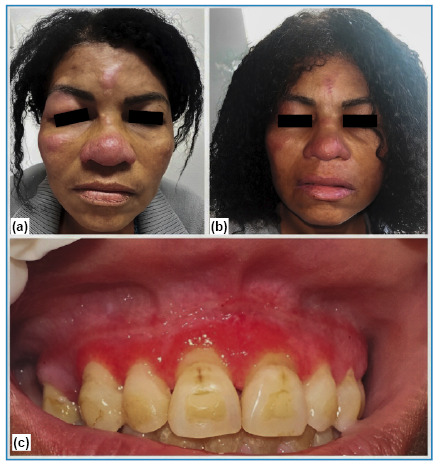
**(a)** Erythematous infiltrated plaques, sarcoid-like, with telangiectasias on the right malar region, nose, forehead, and right upper eyelid. **(b)** Improvement of skin lesions (day 30 of treatment). **(c)** Gingival ulcerated lesion with hemorrhagic dots (moriform stomatitis).

A 50-year-old female farmer from a rural area in Espírito Santo State/Brazil complained of erythematous lesions on her face since 2 years. A previous skin biopsy showed a histological granulomatous pattern without any organisms (hematoxylin and eosin and Fite-Faraco staining). The patient was unsuccessfully treated for granulomatous rosacea, and reported sporadic use of prednisone 20 mg/day. Alcohol and tobacco use were denied. She complained of fever, weight loss (8 kg), a lack of appetite, sialorrhea, oral lesions, headache, and mental confusion. Physical examination revealed sarcoid-like infiltrative erythematous plaques with telangiectasias in the right malar region, nose, forehead, and right upper eyelid, as well as an ulcerated gingival lesion with hemorrhagic dots (Figure 1). Palpation revealed enlarged cervical, anterior, submandibular, and axillary lymph nodes. 

**FIGURE 2:**
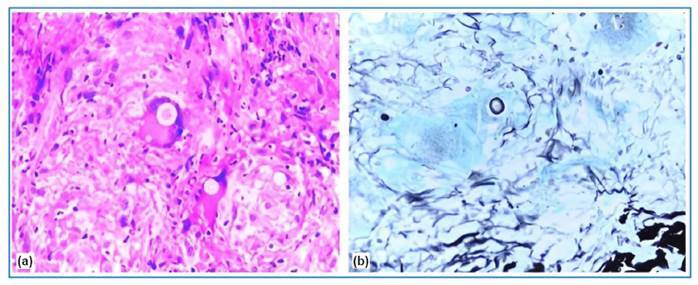
**(a)** Anatomopathological analysis: granulomatous dermatitis with non-sporulating fungi showing thick walls surrounded by multinucleated giant cells of the Langhans type (HE, 400x). **(b)** Anatomopathological analysis: Grocott-Gomori staining demonstrating non-sporulating fungi (Grocott-Gomori, 400x).

**FIGURE 3:**
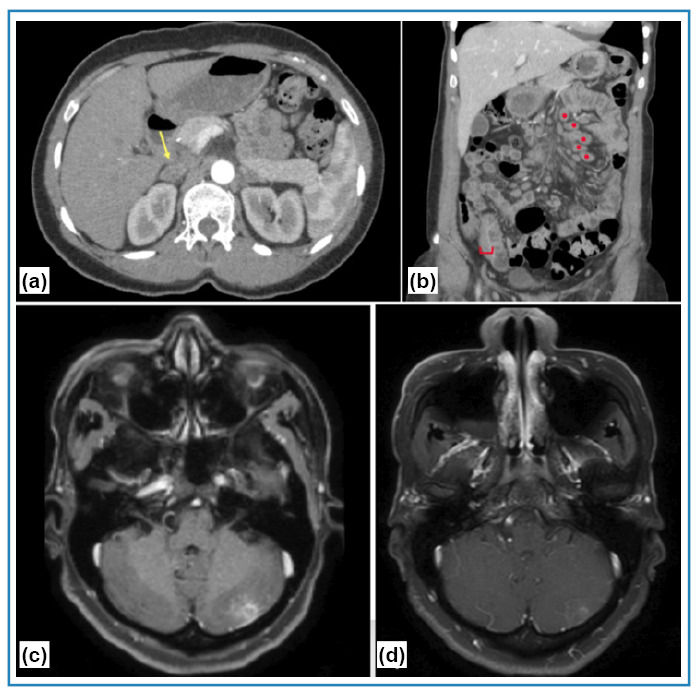
Abdominal Computed Tomography: **(a)** Diffuse adrenal enlargement (yellow arrow); **(b)** Significant parietal thickening of the distal ileum (red bracket) and multiple enlarged lymph nodes in mesenteric chains (red dots). Brain magnetic resonance imaging (MRI): **(c)** Peripheral oval focal lesion in the left cerebellar hemisphere, with ring enhancement by gadolinium, associated with adjacent leptomeningeal impregnation; **(d)** Regression of the oval focal lesion in the left cerebellar hemisphere (day 40 of treatment).

A new skin biopsy revealed granulomatous and suppurative dermatitis and non-sporulating fungi with thick walls, which were stained black by Grocott-Gomori, and were surrounded by Langhans giant cells (Figure 2). The fungal culture results were negative. A double immunodiffusion test using an antigenic preparation for paracoccidioidomycosis showed reactivity up to 1:16. Chest radiography revealed a normal cardiomediastinal silhouette and pulmonary vasculature, and the lungs were clear of focal airspace disease or pleural effusion. Computed tomography showed multiple enlarged lymph nodes in the mediastinal, inguinal, mesenteric, and iliac chains; diffuse bilateral adrenal enlargement; hepatomegaly; mild pericardial effusion; and significant parietal thickening of the distal ileum. Brain magnetic resonance imaging (MRI) revealed a peripheral oval focal lesion in the left cerebellar hemisphere (Figure 3). There were no signs or symptoms of adrenal insufficiency. The results for Venereal disease research laboratory (VDRL), anti-human immunodeficiency virus (HIV), and anti-human T-cell leukemia virus (HTLV) tests were negative. A diagnosis of chronic multifocal PCM manifesting as sarcoid-like skin lesions with severe systemic disease was established, and treatment was initiated with amphotericin B lipid complex at 5 mg/kg/day for 2 weeks, in addition to sulfamethoxazole and trimethoprim at 800/160 mg three times a day. She is still undergoing oral treatment and imaging shows progressive regression of the skin lesions (Figures 1B and 3D). 

## DISCUSSION

Chronic PCM manifests progressively and may occur years after exposure to *Paracoccidioides,* caused by reactivation of the latent endogenous focus of quiescent yeast forms inside granulomas^2^ ^,^ ^4^ ^,^ ^5^. This form is classified as unifocal when one organ or system is impaired, and multifocal, characterized by the involvement of more than one organ such as the adrenal glands, central nervous system, mucous membranes, lymph nodes, or skin, as reported here^3^ ^,^ ^5^ ^,^ ^8^.

Cutaneous involvement in PCM can result in a highly polymorphic spectrum. Lesions may originate from contiguous pre-existing lesions, hematogenous dissemination, lymphatic spread, and, rarely, direct skin inoculation^9^. The morphology and number of lesions depend on the fungal pathogenicity and host immunological response^2^.

Studies have shown that the infiltrative pattern corresponds to up to 26.6% of the observed skin lesions, including sarcoid-type lesions^9^. This clinical presentation is rare and typically manifests as well-defined infiltrated or lichenoid lesions, usually at the cephalic pole^1^. These patients exhibit a predominant T helper 1 (Th1) immune response, which results in compact granulomas with scarce or undetectable fungi. Consequently, histology reveals tuberculoid granulomas with a paucity of fungi, while serology is usually characterized by low titers or negative results. Because of these factors, the disease presents diagnostic challenges and is often misdiagnosed as other granulomatous diseases, such as leishmaniasis, granulomatous rosacea, tuberculoid leprosy, and sarcoidosis^3^. The fungi may present in the pathognomonic form with multiple exosporulation patterns (resembling a "Ship's Wheel" or "Mickey Mouse") or as a non-sporulating form with a thick wall, requiring other diagnostic methods (culture, serology or molecular biology) to identify *Paracoccidioides*, as was done in this study^5^. The presence of moriform stomatitis supported this diagnosis, although it was not pathognomonic.

PCM manifested by sarcoid-like cutaneous lesions typically remains limited to the skin, but the immune response pattern can gradually change owing to the occurrence of adverse events in the individual or the production of virulence factors by the fungus, such as capsule components and exoantigens, which can influence the invasion capacity^2^. These determinants progressively inactivate natural killer cells and CD4+ T cells, leading to a change in the cytokine pattern, predominance of T helper 2 (Th2) response, and increased interleukin (IL)-10, IL-5, and transforming growth factor-β (TGF-β). This process may result in suppression of the macrophage response, which leads to fungal multiplication, granuloma dissolution, and disease spread^10^. The occasional use of corticosteroids, which can lead to probable immunomodulation, in addition to a long untreated disease, may explain why our patient initially presented with limited cutaneous disease and subsequently developed systemic manifestations. 

PCM with sarcoid-like skin lesions associated with systemic involvement, as reported in this case, is rare, with only one case published in literature^9^ and it exhibited less extensive involvement than that in our patient. In conclusion, this case highlights the challenges associated with the diagnosis of PCM presenting with sarcoidosis-like cutaneous lesions, which are rarely mentioned in literature and are likely underdiagnosed. Additionally, we emphasize the importance of PCM as a differential diagnosis for granulomatous diseases because of its high endemicity in South and Central America^5^. 
